# Aging and an Immune Challenge Interact to Produce Prolonged, but Not Permanent, Reductions in Hippocampal L-LTP and mBDNF in a Rodent Model with Features of Delirium

**DOI:** 10.1523/ENEURO.0009-18.2018

**Published:** 2018-06-04

**Authors:** Naoto Tanaka, Giuseppe P. Cortese, Ruth M. Barrientos, Steven F. Maier, Susan L. Patterson

**Affiliations:** 1 Department of Biology, Temple University, Philadelphia, PA 19122; 2 Department of Psychiatry, College of Physicians and Surgeons, Columbia University, New York, NY 10032; 3 Division of Molecular Therapeutics, New York Psychiatric Institute, New York, NY 10032; 4 Department of Psychology & Neuroscience, University of Colorado, Boulder, CO 80309

**Keywords:** BDNF, Delirium, Hippocampus, Interleukin-1beta, LTP, Microglia

## Abstract

Aging increases the risk of abrupt declines in cognitive function after an event that triggers immune system activation (e.g. surgery, infection, or injury). This phenomenon is poorly understood, but rodent models may provide clues. We have previously shown that aging (24-mo-old) F344xBN rats generally do not show significant physical or cognitive impairments. However, their brains mount an exaggerated inflammatory response to signals triggered by a peripheral immune challenge (an intraperitoneal injection of *Escherichia coli* or laparotomy). Their hippocampal levels of the proinflammatory cytokine IL-1β are significantly elevated for at least 8 d, but generally less than 14 d, after infection or surgery. This IL-1β elevation is mirrored by prolonged deficits in a hippocampus-dependent long-term memory task. In contrast, young (3-mo-old) counterparts exhibit only transient elevations in IL-1β that drop to near baseline levels within 24 h. We previously demonstrated that theta burst–evoked late-phase long-term potentiation (L-LTP)—a BDNF-dependent form of synaptic plasticity—is impaired in hippocampal area CA1 of aged animals 4 d after infection. Also, levels of mature brain-derived neurotrophic factor (mBDNF)—the protein isoform required for stabilization of L-LTP—are reduced in hippocampal synaptoneurosomes of aged animals at the same time point. In this study, we investigated whether the deficits in L-LTP and mBDNF persist in parallel with the elevation in IL-1β and impairment in memory. This was the case, consistent with the idea that an exaggerated brain inflammatory response may compromise memory consolidation in part by altering availability of mBDNF to stabilize memory-related synaptic plasticity.

## Significance Statement

Not all cognitive decline is gradual. Older individuals—even those previously healthy and high functioning—are more likely to experience an abrupt decline in mental function (termed delirium) after immune challenge. Even if this is temporary, it is associated with increased risk of ultimately developing dementia. Although clinically important, this phenomenon is much less studied than gradual senescence and aging-associated neurodegenerative disorders. Here we use a naturalistic rodent model to further test the hypothesis that the combination of age and an immune challenge may trigger an exaggerated inflammatory state in the brain, which in turn, disrupts molecular systems critical for memory. These studies may provide mechanistic insights into the earliest stages of inflammation-driven failures of memory-related synaptic plasticity.

## Introduction

When we think about age-related cognitive decline, we tend to think of gradual decrepitude or overt neurodegenerative disease, as in Alzheimer’s disease. However, cognitive decline is not always gradual. Rapid decline can be triggered by activation of the peripheral immune system. Proinflammatory cytokines (e.g., IL-1β and TNF-α) produced by peripheral immune activation can communicate with the brain via both humoral and neural routes, triggering a cascade of effects in the CNS including microglial activation and *de novo* production of proinflammatory cytokines (Maier et al., 2001; Konsman et al., 2002; Dantzer et al., 2008). Interestingly, aging has been shown to sensitize the brain inflammatory response to a variety of experimental immune challenges [e.g., *Escherichia coli*, surgery, lipopolysaccharide (LPS)], increasing the size and duration of the resulting spike in proinflammatory cytokines in the hippocampus (Godbout et al., 2005; Chen et al., 2008; Barrientos et al., 2009).

At 24 mo, Fisher 344/Brown Norway (F344xBN) rats are generally healthy, aging but not senescent. We have previously shown that a single i.p. injection of *E. coli* produces prolonged elevations in IL-1β in the hippocampi of aging 24-mo-old F344xBN rats, but not in 3-mo-old rats (Barrientos et al., 2009). The exaggerated elevation in IL-1β does not impair the initial learning of the test tasks or formation of short-term memories. Instead, it is associated with profound and specific deficits in tasks requiring consolidation of hippocampus-dependent long-term memory (Barrientos et al., 2009). As the levels of IL-1β drop, these deficits fade. Similarly, blocking IL-1β signaling in the brain with an intra-cisterna magna infusion of an IL-1 receptor antagonist (Frank et al., 2010) blocks the memory deficits.

Previously, we examined the effects of age and infection on memory-related synaptic plasticity and levels of hippocampal BDNF (and related proteins) at a single time point, 4 d after the *E. coli* injection. This time point was chosen for several reasons: (1) both the young and aged animals have recovered from the overt symptoms of illness (e.g., fever, loss of appetite, etc.; Barrientos et al., 2006); (2) levels of hippocampal IL-1β are still significantly elevated in the aged rats but have returned to near pre-infection levels in the young rats (Barrientos et al., 2009); and (3) the aged rats show significant deficits in hippocampus-dependent long-term memory, but the young rats do not (Barrientos et al., 2006). We measured a BDNF-dependent, memory-related, long-term form of synaptic plasticity, theta burst–evoked L-LTP in hippocampal area CA1. Deficits in theta-frequency LTP in area CA1 have been shown to distinguish cognitively impaired from unimpaired aged Fischer 344 rats (e.g., Tombaugh et al., 2002). We found that a recent history of infection was associated with reduced theta burst L-LTP in the young rats and that aging greatly exacerbated this effect (Chapman et al., 2010). We also found that levels of mature BDNF (mBDNF, the cleaved protein isoform required for long-lasting forms of memory and LTP; Pang et al., 2004; Barnes and Thomas, 2008) were significantly reduced in the hippocampal synaptoneurosomes prepared from aged rats 4 d after *E. coli* injection (Cortese et al., 2011). Like the deficit in long-term memory (Frank et al., 2010), the deficits in L-LTP and mBDNF could be prevented by interfering with IL-1β signaling in the brain (Chapman et al., 2010; Cortese et al., 2011).

In this study, we extended our examination of theta burst L-LTP and mBDNF to longer time periods (8, 14 and 21 d after infection). The goal was to determine if the deficits in synaptic plasticity and mBDNF would resolve, and if they did, to compare the time courses of their recovery with those of the alterations in IL-1β and hippocampus-dependent long-term memory. The results show that the changes in L-LTP and mBDNF paralleled the changes in IL-1β and memory over time. This suggests that prolonged inflammatory responses in the brain might affect memory-related plasticity of hippocampal synapses, in part by modulating levels of mBDNF and downstream effectors required to stabilize synaptic plasticity.

## Materials and Methods

### Experimental animals

The animals in this study were 3- and 24-mo-old male Fisher 344/Brown Norway F1 hybrid rats from National Institute on Aging Aged Rodent Colony. They were housed in pairs with *ad libitum* access to water and food and were maintained on 12-h light-dark cycle. The animals were allowed to acclimate to the animal facility for a minimum of 10 d before the experiments were begun. All experiments complied with protocols approved by the University of Colorado and Temple University Animal Care & Use Committees.

### The infection model

Stock *E. coli* cultures (15746; ATCC) were thawed and cultured in 40 ml brain–heart infusion (BHI; Difco Laboratories) at 37°C overnight. The number of bacteria in individual cultures was extrapolated from previously determined growth curves. The cultures were centrifuged at 3000 rpm for 15 min, the supernatants were discarded, and the bacterial pellets were suspended in sterile PBS to achieve a final dose of 1.0 × 10^10^ colony-forming units (CFU)/mL in 250 μl. All animals were given an intraperitoneal injection of 250 μl of either *E. coli* or vehicle (sterile PBS).

### Slice preparation

Rats were decapitated, and hippocampi were collected 8 (± 1), 14 (± 1), or 21 (± 1) d after injection of *E. coli* or saline. Experiments on tissue from *E. coli–* or saline-injected animals collected at the different time points were interleaved. Transverse hippocampal slices (400 μm) were prepared employing conventional techniques (Patterson et al., 1992, 1996). Slices were maintained in an interface chamber at 28°C with perfusion of oxygenated artificial cerebral spinal fluid (ACSF; in mm: 124.0 NaCl, 4.4 KCl, 26.0 NaHCO_3_, 1.0 NaH_2_PO_4_, 2.5 CaCl_2_, 1.3 MgSO_4_, and 10 glucose). Slices were left in the chamber to recover for at least 3 h before recording.

### Electrophysiology

Bipolar stimulating (FHC: CBBRC75) and ACSF-filled glass recording (A-M Systems: 603000) electrodes were placed in stratum radiatum to record field excitatory postsynaptic potentials (fEPSPs) from Schaffer collateral–CA1 synapses. All stimuli were set to evoke fEPSP slopes equal to one-third of the maximum in each slice. Test stimuli were delivered every minute, and test responses were recorded for 30 min before starting the experiment to ensure stability. Slices were then tetanized using a theta-burst protocol: 12 bursts of four pulses at 100 Hz, delivered 200 ms apart. The same stimulus intensity was used for tetanization and evoking test responses. Field EPSP recordings were normalized to pre-tetanus baselines and averaged for each group. Error bars indicate SEM. These data were further analyzed by factorial ANOVA, followed by Turkey’s HSD *post hoc* tests (GraphPad Prism 7).

### Synaptoneurosome preparation

Rats were decapitated, and hippocampi were collected 8 or 14 d after *E. coli* or saline injection. Tissue was minced in 500 μl homogenization buffer (HB) with protease and phosphatase inhibitors [in m: 1 Tris, 1 sucrose, 0.5 EDTA, 0.25 EGTA, 0.5 NaF, 1 benzamidine, and 0.1 AEBSF (4-(2-aminoethyl)benzenesulfonyl fluoride)] and homogenized with a glass tissue grinder and a Teflon pestle. The homogenate was centrifuged at 960 × *g* for 15 min to pellet nuclear material and unbroken cells. The remaining supernatant was further centrifuged at 10,000 × *g* for 15 min, yielding an S2 cytosolic supernatant and a P2 crude synaptoneurosomal pellet (pre- and postsynaptic components). The synaptoneurosomal pellet was washed gently in 100 μl HB, homogenized with a 0.5-ml plastic pestle in 100 μl HB and 10 μl of 10× sodium chloride-TRIS-EDTA (1× final concentration), and sonicated. The final P2 fraction obtained using this procedure is enriched for presynaptic and postsynaptic proteins, terminal mitochondria, and cytoplasm and synaptic vesicles (Booth and Clark, 1978; Whittaker, 1993). Synaptic enrichment was confirmed using synaptic markers synaptophysin and postsynaptic density 95 (PSD95). Protein content was determined by the BCA protein assay (Bio-Rad).

### Western blots

All procedures used here have been previously described (Cortese et al., 2011). Synaptoneurosomal samples were denatured in 4× Laemmli buffer and heated at 70°C for 5 min. The resulting protein samples (40 μg each) were loaded onto 4–12% NuPage Bis-Tris SDS-polyacrylamide gels (Invitrogen) and transferred onto polyvinylidene fluoride membranes (Millipore). Membranes were blocked in 5% milk/PBST (PBS with Tween 20) at room temperature for 30 min.

All primary antibodies were incubated overnight at 4°C and then washed 3 × 10 min with PBST. The primary antibodies (and dilutions) used were proBDNF (1:500; ab72440; Abcam), mature BDNF (1:1000; sc-546; Santa Cruz Biotechnology), phospho-TrkB (1:700; pTrkBY816, antisera gift from Moses Chao, New York University School of Medicine, New York, NY) and total (phosphorylated and unphosphorylated) TrkB (1:1000; sc-8316; Santa Cruz Biotechnology), phospho-PLCγ1 (phospholipase C γ1; 1:1000; 07-2134) and total PLCγ1 (1:500; 05–366; Millipore), and phospho-ERK (extracellular response kinase; 1:1000; 9101) and total ERK (1:1000; 9102; Cell Signaling Technology).

To confirm enrichment for synaptic components, blots were probed with synaptic markers, synaptophysin (1:1000; sc-12737; Santa Cruz Biotechnology) and PSD95 (1:1000; United Biomedical). β-Tubulin (1:100,000; MAB1637; Millipore Bioscience Research Reagents) and β-actin (1:5000; sc-47778; Santa Cruz Biotechnology) were used as loading controls. The identity of the BDNF isoform bands in synaptoneurosomes was confirmed by comparison with bands from HeLa cells transfected with a plasmid overexpressing BDNF, producing both the pro- and mature form.

Secondary antibodies were purchased from GE Health care and Bio-Rad and were diluted in the range of 1:5000–1:10,000. Incubations were at room temperature for 1 h, followed by 3 × 10 min washes. Super Signal West Pico Chemiluminescent (Pierce) was applied, and blots were exposed to autoradiography film (Denville Scientific). Blots were then stripped in Restore Western Blot Stripping Buffer (Pierce) for 15 min, washed 3 × 10 min in PBST, and subjected to standard Western blotting conditions.

ImageJ was used to quantify the protein bands, and all bands were normalized to their actin controls. For TrkB, PLCγ, and ERK, the ratio of the phosphorylated form to total expression of each protein was determined. We have previously found that the combined effects of age and infection uniquely disrupt BDNF-dependent memory and synaptic plasticity (Barrientos et al., 2006; Chapman et al., 2010) and also reduce mBDNF (and related proteins) 4–5 d after infection (Cortese et al., 2011). For the protein level time course, we therefore used an unpaired *t* test (GraphPad QuickCals) to determine if the level of the protein of interest in the aged + *E. coli* group differed from the level of the protein in the other groups. The *p* value listed for each protein (or phosphorylation state ratio) is for an unpaired *t* test comparing the mean of the aging + *E. coli* group to the mean of the combined values of the other test groups.

## Results

### Aging and a peripheral immune challenge interact to reduce theta burst–evoked L-LTP

In an earlier report (Chapman et al., 2010), we examined the impact of age and infection on synaptic function at Schaffer collateral-CA1 synapses in hippocampal slices from aged and young rats, 4–5 d after an i.p. injection of *E. coli* or saline. We found no significant differences between groups in basal synaptic transmission or early-phase long-term potentiation (E-LTP). We used two different stimulus protocols to induce late-phase long-term potentiation (L-LTP): either four trains of high-frequency stimulation, which induces robust activation of numerous plasticity-related signaling cascades (Bliss et al., 2007), or a more naturalistic theta burst stimulation, which mimics theta frequency burst firing of CA1 neurons during spatial exploration (O’Keefe, 2007). We found that four-train L-LTP was not significantly affected by age or infection. However, full expression of theta burst L-LTP was suppressed by a recent history of infection, and aging greatly exacerbated this effect.

The immune challenge–evoked elevations in IL-1β and the deficits in long-term memory both last >8 d, but typically <14 d, in aged animals (Barrientos et al., 2009). It seemed plausible that this might also be true of the deficits in theta burst L-LTP.

### Theta burst–evoked L-LTP was still impaired in aged animals 8 d after infection

**Figure 1.**
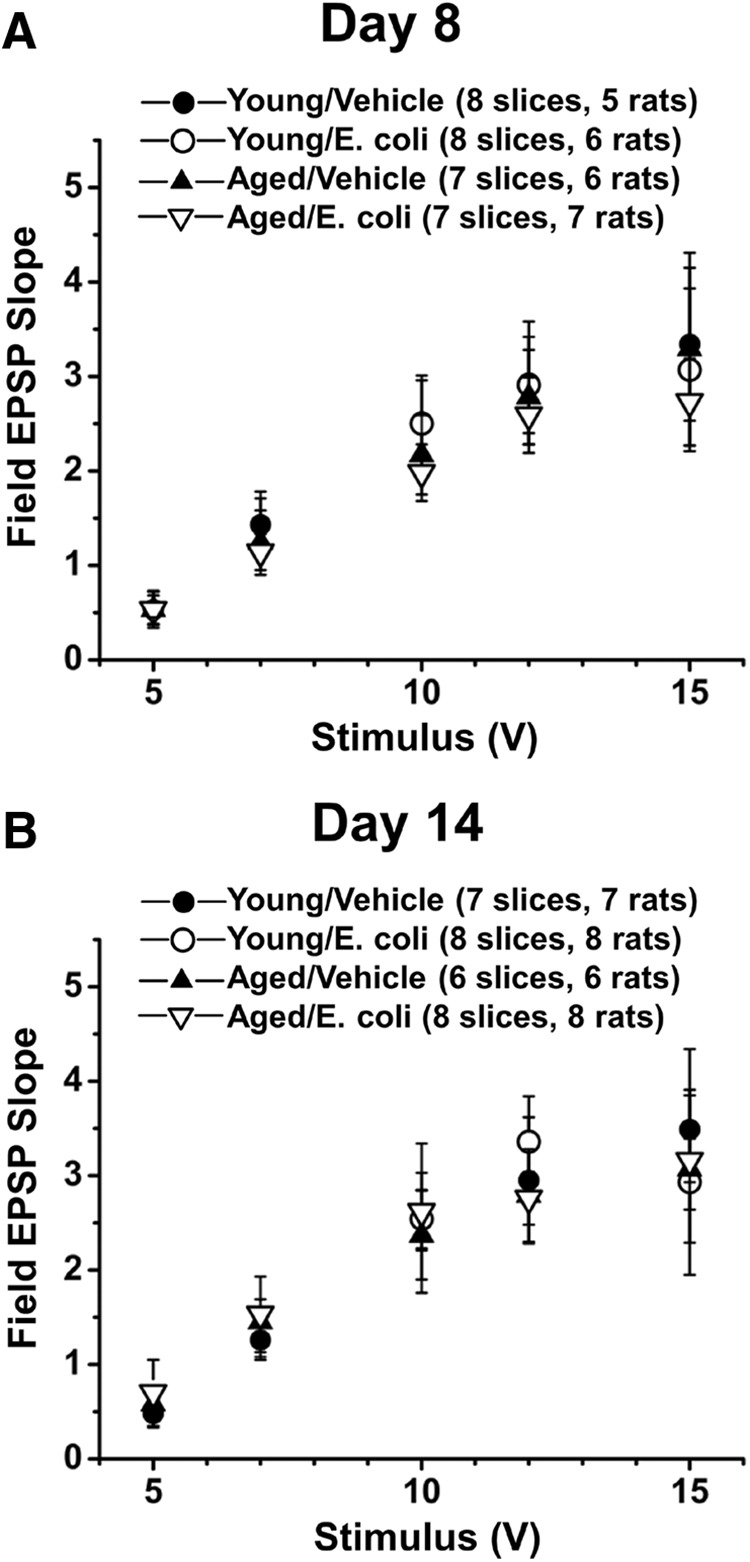
Stimulus–response curves are not altered by age or a history of infection. Plots of fEPSP slopes (in millivolts per millisecond) at various stimulation intensities for hippocampal slices from young and aged rats with and without a recent history of infection show no significant differences in basal synaptic transmission in area CA1. Input-output curves are shown for slice collected 8 d (***A***) and 14 d (***B***) after injection of *E. coli* or saline.

**Figure 2.**
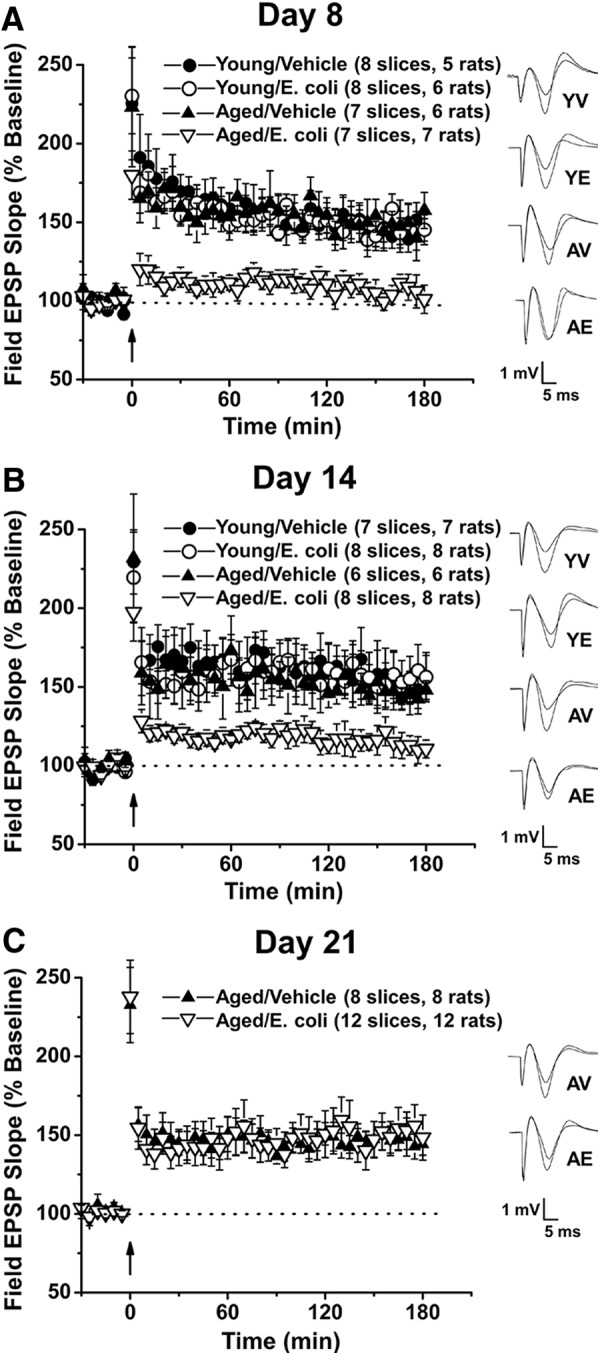
Aging and a peripheral immune challenge interact to produce prolonged, but temporary, reductions in L-LTP. Hippocampal slices were collected from young (3 mo) and aged (24 mo) rats 8, 14, or 21 d after injection of *E. coli* or saline (vehicle). L-LTP was induced in Schaffer collateral-CA1 synapses using theta-burst stimulation (12 bursts of 4 pulses at 100 Hz, delivered 200 ms apart). fEPSP slopes were normalized to pretetanus baselines, averaged, and plotted for each group. Error bars indicate SEM. Insets show representative traces before and 3 h after tetanus. ***A***, L-LTP is severely impaired in slices from aged, but not young, rats 8 d after *E. coli* injection. ***B***, L-LTP is still reduced but recovering in aged rats at 14 d. ***C***, The L-LTP is back to baselines at 21 d.

We examined the impact of age and infection on synaptic function at Schaffer collateral–CA1 synapses in hippocampal slices from aged and young rats, 8 d after an i.p. injection of *E. coli* or saline. As before (Chapman et al., 2010), input-output curves showed no significant difference across the four groups at any stimulating input (*P*_age, infection_ = 0.95 at 5 V, 0.87 at 7 V, 0.85 at 10 V, 0.87 at 12 V, and 0.86 at 15 V; Fig. 1*A*). There was no significant difference in post-tetanic potentiation (immediately after theta burst stimulation) between the groups (*P*_age, infection_ = 0.4130; young/vehicle = 216.9 ± 38.2%, young/*E. coli* = 230.3 ± 24.1%, aged/vehicle = 223.2 ± 32.5%, and aged/*E. coli* = 179.9 ± 15.4%; Fig. 2*A*). However, theta burst L-LTP was still severely impaired in aged rats 8 d after infection (*P*_age, infection_ = 0.0070; percentage baseline 3 h after tetanus: young/vehicle = 145.3 ± 9.5%, young/*E. coli* = 145.2 ± 9.1%, aged/vehicle = 157.5 ± 11.5%, and aged/*E. coli* = 101.0 ± 9.0%; Fig. 2*A*). Turkey’s *post hoc* tests supported this statistical analysis, showing significance when the aged *E. coli* group was compared to young saline (*p* = 0.0101), young *E. coli* (*p* = 0.0180), or aged Saline (*p* = 0.0021).

### Theta burst-evoked L-LTP was recovering in aged animals 14 d after infection

The effects of age and infection were subtler at 14 d. Input-output curves indicated no significant difference across the four groups (*P*_age, infection_ = 0.77 at 5 V, 0.78 at 7 V, 0.73 at 10 V, 0.60 at 12 V, and 0.66 at 15 V; Fig. 1*B*). There was no significant effect on posttetanic potentiation (*P*_age, infection_ = 0.8091; young/vehicle = 229.4 ± 20.4%, Young/*E. coli* = 209.2 ± 32.9%, aged/vehicle = 231.7 ± 40.6%, and aged/*E. coli* = 197.4 ± 18.3%; Fig. 2*B*). The initial statistical analysis of L-LTP revealed no significance in the combined effects of age and infection (*P*_age, infection_ = 0.0757; percentage baseline 3 h after tetanus: young/vehicle = 155.4 ± 14.6%, young/*E. coli* = 156.2 ± 14.4%, aged/vehicle = 148.0 ± 5.9%, and aged/*E. coli* = 110.6 ± 5.7%; Fig. 2*B*). However, the group means and graphs suggest some remaining reduction in the L-LTP of aged *E. coli* animals; Tukey’s *post hoc* tests indicated that the aged *E. coli* group differed from young saline (*p* = 0.0169) and young *E. coli* (*p* = 0.0220). This was not the case when the aged *E. coli* group was compared to the aged saline group (*p* = 0.0542). Together, these results suggest significant, but incomplete, recovery in the capacity for L-LTP at 14 d.

### Theta burst–evoked L-LTP had returned to control levels in aged animals 21 d after infection

We extended our investigation to 21 d to determine if the *E. coli* evoked deficits in L-LTP in the aged rats would fully resolve (Fig. 2*C*). There was no significant difference in post-tetanic potentiation at 21 d (aged/vehicle vs. aged/*E. coli*: *p* = 0.9081; aged/vehicle = 232.7 ± 23.9%, and aged/*E. coli* = 237.8 ± 23.3%). The results show normal levels of L-LTP in aged E. coli animals at this time point (aged/vehicle vs. aged/*E. coli*: *p* = 0.8232; percentage baseline 3 h after tetanus: aged/vehicle = 144.2 ± 6.9%, and aged/*E. coli* = 148.4 ± 14.3%).

### Levels of the mature BDNF protein isoform were significantly reduced in hippocampal synaptoneurosomes prepared from aged rats 8 d, but not 14 d after infection

The forms of long-lasting memory and synaptic plasticity compromised by age and infection are highly dependent on BDNF (Tyler et al., 2002; Chao, 2003; Lu, 2003; Bramham and Messaoudi, 2005). We have therefore hypothesized that aging and infection might compromise production or processing of BDNF protein, reducing the availability of BDNF for memory-related plasticity processes at synaptic sites (Cortese et al., 2011). BDNF is synthesized as a precursor, proBDNF, which undergoes post-translational cleavage to produce mature BDNF (mBDNF), the protein isoform required for long-lasting forms of memory and LTP (Pang et al., 2004; Barnes and Thomas, 2008).

**Figure 3.**
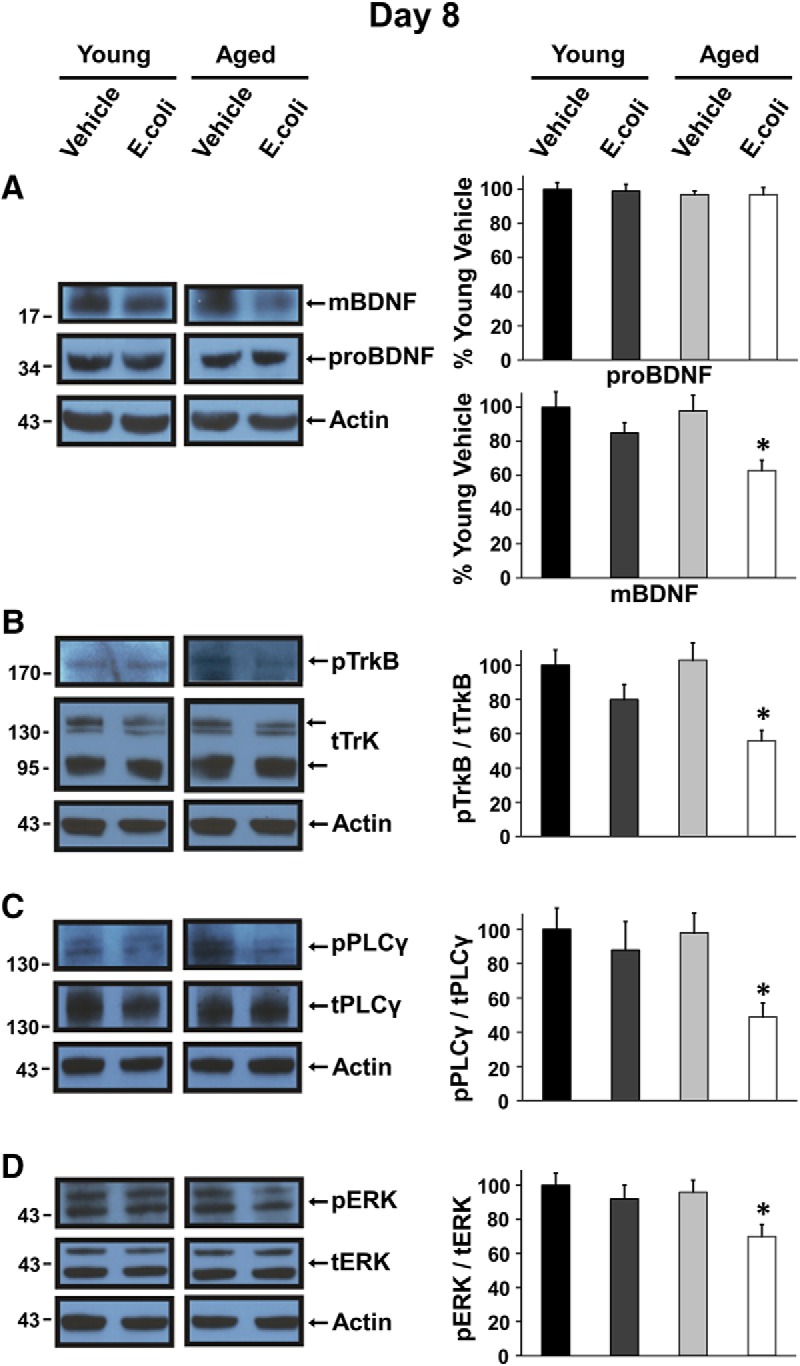
Levels of the mature BDNF protein isoform and activation of TrkB and downstream signaling are reduced in aged rats 8 d after infection. Western blot analysis was conducted using hippocampal synaptoneurosomes prepared from young and aged rats 8 d after injection of *E. coli* or vehicle; representative examples are shown. ***A***, levels of mBDNF, but not proBDNF, are diminished in the aged animals 8 d after infection. ***B–D***, Graphs present the ratio of phosphorylated TrkB (pTrkB), PLCγ1 (pPLCγ), or ERK (pERK) to total expression of TrkB (tTrkB), PLCγ1 (tPLCγ), or ERK (tERK). Levels of phosphorylated TrkB (***B***), PLC γ1 (***C***), and ERK (***D***) are significantly lower in the aged animals 8 d after infection. Protein bands were quantified using ImageJ, normalized to their actin controls, and expressed as percentages of mean protein levels from young vehicle-injected animals. Error bars represent SEM. All graphs here and below represent at least three independent experiments, with 1–2 animals per group in each experiment.

**Figure 4.**
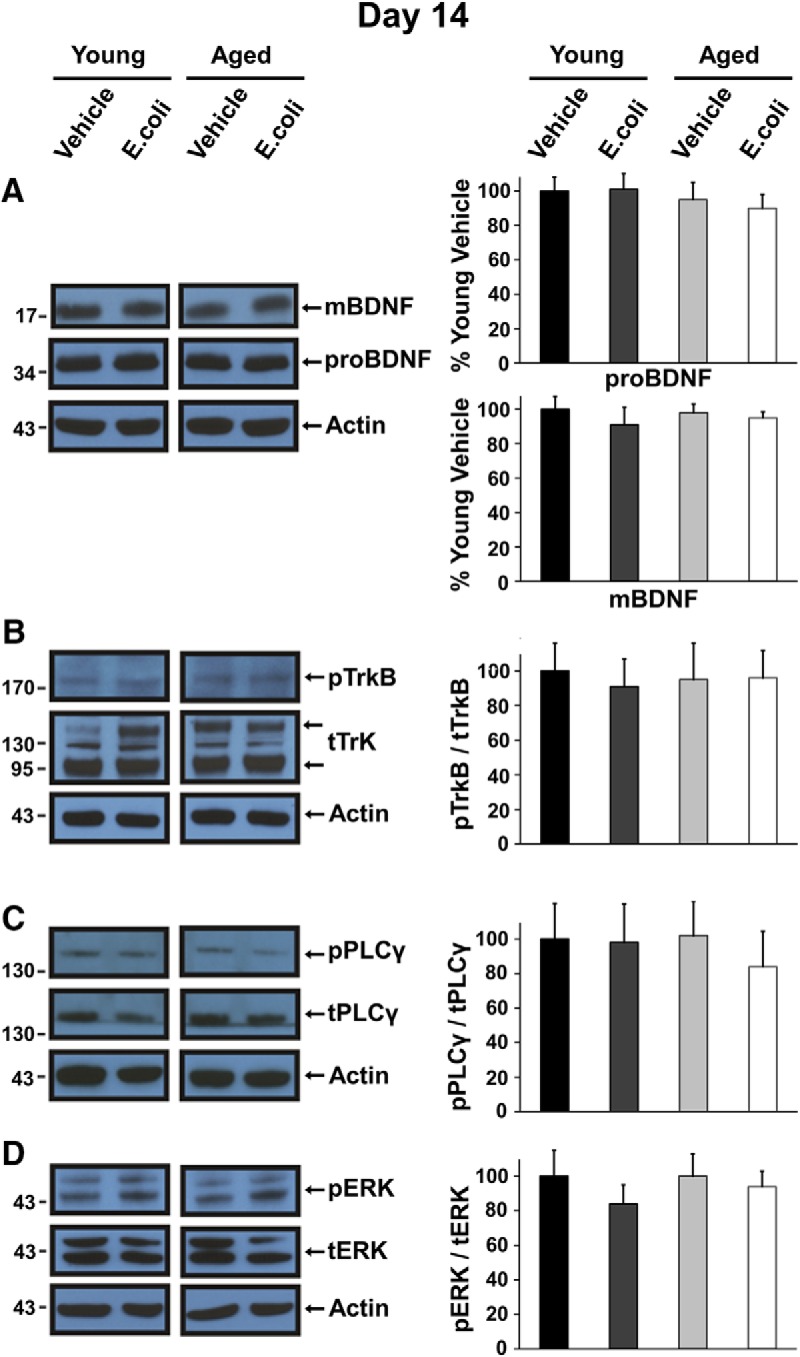
Levels of mBDNF and activation of TrkB, PLCγ1, and ERK in aged rats have returned to normal 14 d after *E. coli* injection. Western blot analysis of hippocampal synaptoneurosomes prepared from young and aged rats 14 d after injection of *E. coli* or vehicle; representative examples are shown. ***A–D***, Aged *E*. *coli* animals show no significant difference in levels of mBDNF (***A***) or activation of TrkB (***B***), PLC γ1 (***C***), or ERK (***D***). Quantification was as described in the legend for Fig. 3.

We previously demonstrated that levels of the mBDNF protein were significantly reduced in synaptoneurosomes prepared from the hippocampi of aged rats 4–5 d after injection of *E. coli* (Cortese et al., 2011). In this study, we examined later time points—8 and 14 d after injection—to determine if levels of mBDNF in aged animals would recover as levels of IL-1β dropped toward pre-infection baselines (Barrientos et al., 2009). Western blot analysis with an antibody against the mature domain of BDNF (Lee et al., 2001) supported this hypothesis. At 8 d, levels of mBDNF (Fig. 3*A*) were still significantly reduced in hippocampal synaptoneurosomes from the aged *E. coli* group compared to the other groups (*t*_(18)_ = 2.3427, *p* = 0.0308). In contrast, levels of mBDNF were back to normal at 14 d (Fig. 4*A*), showing no statistical significance (*t*_(14)_ = 0.0936, *p* = 0.9267). Meanwhile, Western blot analysis with an antibody against a specific proBDNF signal revealed no significant difference at 8 d (Fig. 3*A*; *t*_(14)_ = 0.1983, *p* = 0.8457) or 14 d (Fig. 4*A*; *t*_(10)_ = 0.4676, *p* = 0.6501).

### Age and infection interact to reduce activation of TrkB and downstream signaling systems, but this effect is not permanent

Mature BDNF binds to the tropomyosin-related kinase B receptor (TrkB), triggering a cascade of phosphorylation events, starting with the receptor, which can activate downstream signaling pathways including the phospholipase C-γ1 (PLCγ1) and the Ras/extracellular signal-regulated kinase (ERK) pathways (Patapoutian and Reichardt, 2001; Segal, 2003). These pathways ultimately lead to the transcription and translation events required for L-LTP (Finkbeiner et al., 1997; Minichiello, 2009).

We previously reported significantly reduced levels of mBDNF in synaptoneurosomes prepared from the hippocampi of aged rats 4–5 d after injection of *E. coli* (Cortese et al., 2011). Consistent with the decreased availability of mBDNF, we also found significantly reduced activation of TrkB and the PLCγ1 and ERK downstream signaling pathways (Cortese et al., 2011). Here, we examined the impact of age and infection on activation of TrkB and downstream signaling 8 and 14 d after injection.

Analysis of Western blots using an antibody against phosphorylated TrkB (pTrkB) and an antibody against total TrkB (tTrkB; sc-8316 antibody; Santa Cruz Biotechnology) showed that the ratio of pTrkB/tTrkB was significantly reduced in synaptoneurosomes from aged *E. coli* rats at 8 d compared to the other groups (Fig. 3*B*; *t*_(10)_ = 2.2692, *p* = 0.0466). At 14 d (Fig. 4*B*), levels of phospho-TrkB were back to baselines in the aged *E. coli* (*t*_(10)_ = 0.0470, *p* = 0.9635).

Activation of PLCγ1 and ERK was also examined, and these results were consistent with the changes in levels of mBDNF and activation of TrkB. The ratio of phosphorylated PLCγ1 (pPLCγ1; 07-2134 antibody; Millipore) to total PLCγ1 (tPLCγ1; 05-366 antibody; Millipore) was significantly reduced in synaptoneurosomes of aged *E. coli* injected rats at 8 d (Fig. 3*C*; *t*_(14)_ = 2.1706, *p* = 0.0477), but was back to baselines at 14 d (Fig. 4*C*; *t*_(10)_ = 0.5000, *p* = 0.6279). The ratio of phosphorylated ERK (pERK; 910 antibody1; Cell Signaling) to total ERK (tERK; 9102 antibody; Cell Signaling) was profoundly reduced in synaptoneurosomes of aged *E. coli* injected rats at 8 d (Fig. 3*D*; *t*_(18)_ = 2.5149, *p* = 0.0216) but back to normal at 14 d (Fig. 4*D*; *t*_(10)_ = 0.0047, *p* = 0.9964).

## Discussion

We have previously demonstrated that in 24-mo-old F344xBN rats, a single i.p. injection of *E. coli* triggers an exaggerated hippocampal production of IL-1β (Barrientos et al., 2009) that is associated with profound deficits in contextual fear conditioning, a hippocampus-dependent memory task (Barrientos et al., 2006), in theta burst–evoked L-LTP (Chapman et al., 2010), and in mBDNF/TrkB signaling (Cortese et al., 2011). Blunting the effects of IL-1β in the brains of aged animals using the IL-1 receptor antagonist IL-1Ra blocks all of these deficits (Chapman et al., 2010; Frank et al., 2010; Cortese et al., 2011). We have also determined that the elevation in IL-1β and the associated memory deficits do subside, but slowly—they last more than a week, but typically <2 weeks (Barrientos et al., 2009). Here we extend these observations, further exploring the strength of these correlations over time and asking if the infection-induced deficits in theta burst L-LTP and mBDNF will also subside and follow the same time course of recovery as the alterations in IL-1β and memory.

Our principle new findings are that (1) theta burst L-LTP remained profoundly compromised in aged animals 8 d after *E. coli* injection, but the much milder suppression observed in young animals 4–5 d after injection (Chapman et al., 2010) had resolved; there were also still significant deficits in mBDNF levels and signaling in the aged animals at this time point; (2) theta burst LTP in the aged animals showed significant, but incomplete, recovery 14 d after the *E. coli* injection, and mBDNF levels and signaling in aged animals were no longer significantly impaired; and (3) 21 d after the *E. coli* injection, theta burst LTP in the aged animals had completely recovered. Thus, the exaggerated elevation of IL-1β is precisely mirrored by the deficits in memory and in mBDNF/TrkB signaling: as levels of hippocampal IL-1β decline, memory and mBDNF/TrkB signaling recover. There was a slight lag in the full recovery of theta burst L-LTP, consistent with reports of a critical threshold level of BDNF being required to set the conditions necessary for full expression of BDNF-dependent forms of long-lasting synaptic plasticity such as theta burst L-LTP (Korte et al., 1995, 1996; Patterson et al., 1996; Pang et al., 2004).

**Figure 5.**
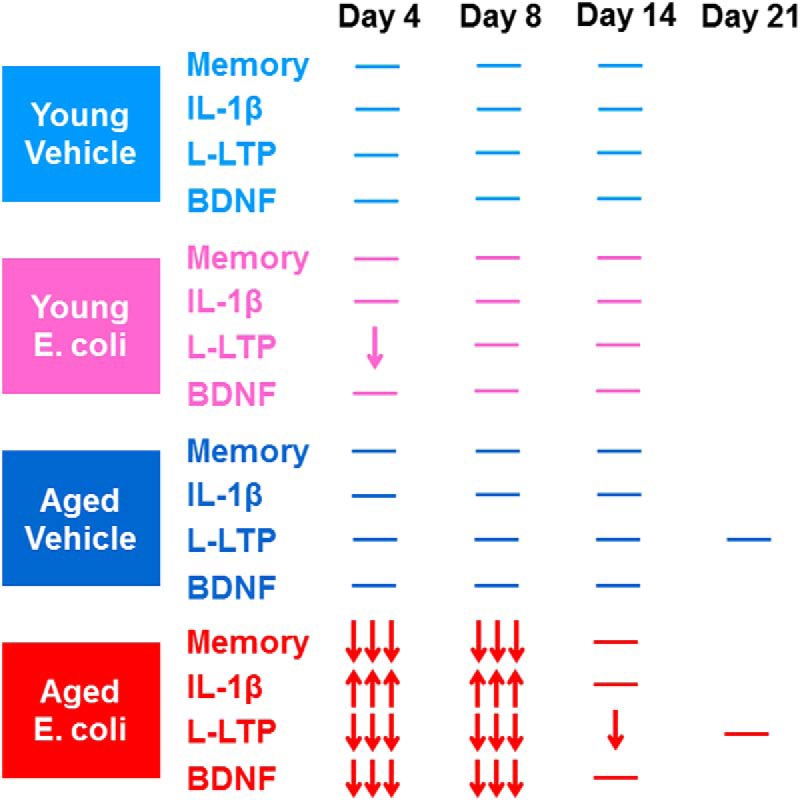
A summary of the effects of age and infection at multiple time points after injection of *E. coli* or saline. *Memory* = hippocampus-dependent long-term memory; *IL-1β* = levels of proinflammatory cytokine interleukin-1β in hippocampal synaptoneurosomes; *L-LTP* = theta burst–evoked L-LTP in the hippocampal CA1 area; and *BDNF* = levels of mature BDNF and activity of related proteins in hippocampal synaptoneurosomes. Upward arrows indicate an increase, and downward arrows show a reduction. Three arrows represent severe deficits, and one arrow means impairments to a lesser degree. Horizontal lines indicate baseline values. Data summarized for day 4 are drawn from earlier publications: *Memory and IL-1β* (Barrientos et al., 2006, 2009), *L-LTP* (Chapman et al., 2010), and *BDNF* (Cortese et al., 2011).

Taken together, these results (summarized in Fig. 5) are consistent with the hypothesis that the exaggerated hippocampal inflammatory response produced by age and an infection might decrease availability of BDNF at hippocampal synapses, and thus contribute to deficits in forms of long-lasting memory and synaptic plasticity that require BDNF for their complete expression.

Many studies have examined the effects of normal aging on memory, synaptic plasticity, and BDNF signaling, with varied results. It is now recognized that variability in cognitive and synaptic functioning, and in BDNF signaling, increases with increasing age in individuals and populations. Aging is frequently, though not always, associated with some degree of cognitive decline and with disruptions in related forms of synaptic plasticity; there appears to be considerable variability depending on the experimental protocols used and the species, strains, and ages of the subjects (Landfield and Lynch, 1977; Gage et al., 1984; Barnes and McNaughton, 1985; Deupree et al., 1991; Diana et al., 1995; Gallagher et al., 2003; Tombaugh et al., 2005; Lynch et al., 2006; Sharma et al., 2015). Data from human autopsy studies and animal models examining variability in cognitive functioning with aging suggest that when deficits in hippocampus-dependent memory occur, they do not arise from a loss of hippocampal neurons, or initially even from a loss of synapses, but rather from more subtle alterations in synaptic efficacy (Lister and Barnes, 2009). For example, in hippocampal area CA1, the basic mechanisms for synaptic modification persist in old age, but the threshold for producing long-lasting, memory-related plasticity increases. High-frequency stimulation protocols can still induce L-LTP, but milder, more naturalistic types of stimulation are less likely to do so (Lynch et al., 2006). Because BDNF is a key mediator of synaptic efficacy in circuits critical for cognition (Bramham and Messaoudi, 2005; Leal et al., 2015), it has long been suspected that disruption of BDNF signaling systems might play a significant role in aging-associated cognitive decline. Somewhat surprisingly, it now appears that basal levels of BDNF and its receptor TrkB in the brain do not change very much as a result of aging alone (Pang and Lu, 2004), although significant changes are seen in some neurodegenerative diseases (Zuccato and Cattaneo, 2009).

Clearly, age is not the only important variable in age-related cognitive decline. The present results add to a growing body of evidence suggesting that much of this variability arises from complex interactions of age with genetics, lifestyle, and life history. Aging sensitizes the brain immune response (Godbout et al., 2005; Chen et al., 2008; Barrientos et al., 2009), and increases the vulnerability of systems for memory-related plasticity to immune challenges. This may represent an important source of variability in cognitive function in the aging brain.

Aberrantly elevated levels of proinflammatory molecules such as IL-1β can compromise memory and synaptic plasticity. Interleukin-1β, its receptor, and the natural IL-1 receptor antagonist are all present at relatively high levels in the hippocampus (Lechan et al., 1990; Takao et al., 1990; Ban et al., 1991). This expression pattern suggests that IL-1 signaling may play a significant role in modulating hippocampal functions, and that memory-related plasticity processes in the hippocampus may be particularly vulnerable to dysregulated IL-1 signaling. This may be particularly true in aging, since sensitivity to IL-1β is augmented in aged hippocampal synapses (Prieto et al., 2015). Low basal levels of IL-1β appear to be required for long-term memory and synaptic plasticity in the healthy hippocampus (Yirmiya and Goshen, 2011). However, performance on hippocampus-dependent memory tasks can be seriously compromised by manipulations that result in too much IL-1β. These include intraventricular (Oitzl et al., 1993) and hippocampal (Barrientos et al., 2002) IL-1 administration, multiweek elevation of IL-1β in the hippocampi of transgenic mice (Hein et al., 2010), and elevation of endogenous IL-1β evoked by infections (Gibertini et al., 1995; Barrientos et al., 2006; Chen et al., 2008) or psychological stressors (Pugh et al., 1999). Similarly, in young rodents, experimental elevation of IL-1β can block full expression of LTP in several areas of the hippocampus (Lynch, 2010). Application of high levels of IL-1β to rodent hippocampal slices reduced LTP in areas CA1 (Bellinger et al., 1993; Ross et al., 2003) and CA3 (Katsuki et al., 1990) and in the dentate (Cunningham et al., 1996; Coogan and O’Connor, 1997). *In vivo* LTP in the dentate was inhibited by intraventricular injection of IL-1β (Murray and Lynch, 1998; Kelly et al., 2003) or an i.p. injection of LPS, a potent endotoxin that triggers strong immune responses (Lynch, 2004; Barry et al., 2005).

The memory and plasticity processes compromised by excess IL-1β are highly dependent on BDNF, and there is increasing evidence that experimental elevation of proinflammatory cytokines in the brain can diminish the availability of BDNF—potentially from both neuronal and microglial (Parkhurst et al., 2013) sources—for memory-related processes (Patterson, 2015). Infusing IL-1β into the hippocampus decreases its capacity for transcription of BDNF after fear learning (Barrientos et al., 2004), while infusion of IL-1Ra protects it during social isolation stress (Barrientos et al., 2003). Perhaps not surprisingly, intraperitoneal injection of high levels of IL-1β or LPS produced an acute (within 4 h) reduction in hippocampal BDNF mRNA (Lapchak et al., 1993). However, expression of specific activity– and plasticity-associated BDNF mRNA transcripts, and the capacity to recruit these transcripts after fear learning, was still reduced in the hippocampi of aged rats 4 d after i.p. *E. coli* (Chapman et al., 2012). Aberrantly elevated levels of cytokines also appear to compromise production of the BDNF protein and downstream signaling. Levels of BDNF protein in the hippocampus showed a dose-dependent reduction 7 h after i.p. LPS (Guan and Fang, 2006). A high dose of LPS injected i.p. into young mice is reported to produce a small (15%) reduction in both proBDNF and mature BDNF in brain synaptoneurosomes 3 d later (Schnydrig et al., 2007). Intraperitoneal injection of *E. coli* produces a large reduction in mBDNF and TrkB signaling in hippocampal synaptoneurosomes from aged rats 4–5 d later (Cortese et al., 2011). There are also indications that excessive IL-1β may sometimes interfere with the neuroprotective effect of BDNF-induced signal transduction, in addition to compromising its plasticity-related functions (Tong et al., 2008, 2012; Cortese et al., 2011; Chapman et al., 2012).

The hypothesis that exaggerated brain inflammatory responses might disrupt BNDF-dependent synaptic plasticity and neuroprotective processes has broad implications for understanding, preventing, or treating cognitive dysfunction in a variety of disorders associated with neuroinflammation or dysregulated brain immune responses, but may be particularly informative in the context considered here. Very few studies have focused on the mechanisms of acute cognitive decline (termed delirium) following an inflammatory event, despite its clinical prevalence and association with markedly increased risk of progression to and acceleration of dementia (Fong et al., 2009; Cunningham, 2011). The immune challenge–triggered cognitive decline we model here in rodents resembles that observed in human delirium. There is a common aging-associated vulnerability, and the pathology shares a similar trajectory and time course; there may well be elements of a common etiology.

## Synthesis

Reviewing Editor: Alfonso Araque, University of Minnesota Decisions are customarily a result of the Reviewing Editor and the peer reviewers coming together and discussing their recommendations until a consensus is reached. When revisions are invited, a fact-based synthesis statement explaining their decision and outlining what is needed to prepare a revision will be listed below. The following reviewer(s) agreed to reveal their identity: Jonathan Godbout, Sreedharan Sajikumar

The reviewers have found merit and interest on the findings reported. I concur with the reviewers' comments expressing the interest of the study. However, they also expressed some concerns about several issues that need to be addressed and clarified. I also consider pertinent the concerns expressed. We believe that addressing those concerns, which may require less than 2 months' work and will definitely improve the manuscript, is necessary to firmly validate the results.

Specific comments of the reviewers:

Reviewer 1.

The combination of age and an immune challenge trigger an exaggerated inflammatory state in the brain, which in turn disrupts memory-related synaptic plasticity. This mechanism is important for understanding inflammation-driven failures in memory-related synaptic plasticity. The authors, Tanaka et al., used electrophysiology and protein assays to determine the effects of age and E. coli infection on long-term potentiation (LTP). This manuscript, titled "Aging and an immune challenge interact to produce prolonged, but not permanent reductions in hippocampal L-LTP and mBDNF in a rodent model with features of delirium", follows up with a previous paper examining these effects at 4-5 days post-infection, continuing their studies to 8 and 14 days post-infection. These data suggest the deficits in late-phase LTP (L-LTP) and mBDNF/TrkB signaling persist at 8 days post-infection in aging mice, but they are (for the most part) resolved by 14 days post-infection.

1. Along with the main effects and interactions for the electrophysiology data, please provide the results of the post-hoc analysis. Additionally, the insignificance in Figure 1B seems to come from increased error in the Aged/Vehicle group, as all other means do not seem to change from Day 8 to 14. Treating time as a variable may increase the power of this analysis (show L-LTP remains depressed until Day 21).

2. More statistics are required for Western blot analysis. The methods state, "The p value listed for each protein (or phosphorylation state ratio) is for an unpaired t test comparing the mean of the aging + E. coli group to the mean of the summed values of the other test groups." First, the word "summed" should be substituted with "combined". Second, ANOVA and post hoc comparisons should be performed to ensure the combined groups are not significantly different from one another if this combination is indeed to be performed. This approach also calls into question why a standard ANOVA comparison is not being performed to ensure the difference is not due to a main effect of age that is lost when combining two young groups with one aging group. Moreover, the statistics reported for Figure 3D do not seem correct. It is claimed that the levels of pERK are "back to normal" with p = 0.0047, which indicates a highly significant difference.

3. Why are Adult animals omitted from the Day 21 electrophysiology? This changes the statistical analyses between time-points.

4. The Results subsections for electrophysiology could be combined into a single subsection to make result presentation more fluid.

5. The Western blot bar graph legends do not fit the graphs if the bar colors are all different. The pro- vs. m-BDNF bars should also be separated, as the comparison being highlighted in this figure is pro- vs. m-BDNF, not age or infection as it should be.

6. The manuscript contains several typographical errors and inconsistencies that should be addressed (e.g. "tetanous", Molar = "M" not "m", "synaptic makers", "interact to reductions", "infecxtion", "significance effect", "key mediatory").

Reviewer 2.

The study by Tanaka et al., show that brain inflammatory response elicited by intraperitoneal injection of E. coli has an effect on long-term plasticity in aging and is dependent on the availability of mBDNF.

The study is interesting and well designed. The authors should address the following points to improve the impact of their studies:

1) It has been reported earlier that microglial BDNF increases neuronal TrkB phosphorylation (PMID: 2436028), thus it would helpful if the authors should specify whether the BNDF changes observed in their studies after intraperitoneal injection of E. coli is due to microglia activation or due to neuronal changes.

2) The authors show that the magnitude of TBS- LTP is identical in young and aged vehicle (Figure 1 A & B). There are many studies reported earlier the depleted level of BDNF with aging thus the authors should observe a decrease in the magnitude of TBS-LTP. Indeed , using strong tetanization protocol it has been demonstrated earlier that the magnitude of late- LTP is less in aged rats compared to young , in addition synaptic tagging and capture processes were also impaired (PMID: 26577291 , PMID: 28065806). It should be helpful for the readers to include this point in the discussion.

3) The authors stated that they did not observe any changes in the basal synaptic responses in young and aged animals. Interestingly the analog traces show drastic changes in the presynaptic (fibre) volley. For instance in Figure. 1 A and B group YV and AL groups. These differences provide an indication that stimulation intensities were not within a comparable range. It would be helpful to provide a graph showing input -output relations of different groups to further confirm that the basal synaptic transmission were identical in all groups.
